# Transcriptomic and Proteomic Analyses of the Liver and Ileum Identify Key Genes and Pathways Associated with Low and High Groups of Social Genetic Effect of Residual Feed Intake

**DOI:** 10.3390/ani15091345

**Published:** 2025-05-07

**Authors:** Patrick Kofi Makafui Tecku, Zhenjian Zhao, Kai Wang, Xiang Ji, Dong Chen, Qi Shen, Yang Yu, Shengdi Cui, Junge Wang, Ziyang Chen, Jia Xue, Guoqing Tang

**Affiliations:** 1 Key Laboratory of Livestock and Poultry Multi-Omics, Ministry of Agriculture and Rural Affairs, College of Animal Science and Technology, Sichuan Agricultural University, Chengdu 611130, China; ptecku@gmail.com (P.K.M.T.); 0freestyle9@sina.com (Z.Z.); wangkai70@newhope.cn (K.W.); 18338309558@163.com (X.J.); xiangyao2kexiyou@163.com (D.C.); qi.shen@ugent.be (Q.S.); 18200329202@163.com (Y.Y.); cui313164192@163.com (S.C.); wjgdms@163.com (J.W.); czy84719@163.com (Z.C.); 2 Farm Animal Genetic Resources Exploration and Innovation Key Laboratory of Sichuan Province, Sichuan Agricultural University, Chengdu 611130, China; 3 Key Laboratory of Digital Intelligent Breeding Technology Innovation for Swine and Poultry, Ministry of Agriculture and Rural Affairs, New Hope Liuhe Co., Ltd., Chengdu 610023, China; 4 Chengdu Animal Disease Prevention and Control Center, Chengdu 610041, China; lizsit@163.com

**Keywords:** social genetic effect, feeding behavior, residual feed intake, pig, key gene

## Abstract

Social genetic effects (SGEs) refer to how the genotypes of other individuals impact an individual’s phenotype within a population. These effects significantly influence the feeding behavior and production performance in pigs, though their mechanisms are not well understood. This study examined two pig groups with extreme SGE values for residual feed intake (RFI), analyzing their feeding behavior and the molecular mechanisms involved using transcriptomics and proteomics analysis of liver and ileum tissues. Pigs with higher SGE values exhibited distinct feeding patterns, spending more time at the feeder but making fewer visits. They consumed less overall feed but had a higher intake per visit. Differentially expressed genes and proteins were identified in the liver and ileum and were associated with processes such as mitochondrial functions, oxidative phosphorylation, and cholesterol metabolism. Integrated analysis supported these findings. Combined transcriptome and proteome analysis identified potential key genes that were associated with processes including mitochondrial processes, oxidative phosphorylation, fat digestion and absorption, and cholesterol metabolism. The results showed that pigs with differing SGE values display different feeding behaviors and utilize distinct molecular pathways affecting RFI. These findings offer valuable insights into how SGEs influence feed efficiency and shed light on the fundamental mechanisms underlying it.

## Simple Summary

Social genetic effects (SGEs) are how the genes of other animals affect an individual’s traits, such as the feed efficiency within a group. This study investigated the processes involved in SGEs related to the residual feed intake (RFI) in pigs. Pigs with high and low SGE values for RFI were selected, and their feeding behavior, growth performance, and gene and protein expression in the liver and ileum were analyzed. The study found that pigs with higher SGE values had unique feeding habits, spending more time at the feeder but consuming less food overall. Specific genes and proteins were identified: genes in the liver were linked to energy processes, while those in the ileum were related to fat digestion and cholesterol metabolism. These results suggest that pigs in different SGE groups have different feeding behaviors and use various biological pathways to manage feed intake. This research could help improve feed efficiency in pig production.

## 1. Introduction

An individual’s phenotype is shaped by its genes, the environment, and their interactions. While the genetic variation of an individual’s phenotype is primarily determined by its genotype, it is also influenced by gene expression interactions with other individuals [1]. The direct influence of an individual’s own genotype on its phenotype is known as direct genetic effects (DGEs), whereas the influence of other individuals’ genotypes (typically conspecifics) on that phenotype is called social genetic effects (SGEs) [2]. Social genetic effects often manifest through interactions between individuals within a group, such as social dominance, intraspecific competitive ability, and mating systems in animals [3]. In plants, SGEs are observed in ecological interactions, where traits such as plant size and fitness are influenced by neighboring plants [4,5]. With the advancement of linear model analysis methods in recent years, the estimation of SGEs has become easier and more accessible [6,7]. Applying best linear unbiased prediction (BLUP) models that incorporate SGEs allows for the evaluation of the SGEs of a trait, reflecting the influence of an individual’s genotype on other individuals. For most traits, the SGEs’ variance accounts for 9–98% of the total genetic variance [8]. Baud et al. reported that SGEs explain up to 29% of phenotypic variance, and their contribution exceeds that of DGEs for several health and disease traits in mice [9]. Studies have shown that the use of traditional breeding models that do not consider the SGEs among individuals has led to negative selection responses in terms of body weight and mortality rate in Japanese quails [10]. Also, pigs reared in captivity may exhibit harmful behaviors such as ear biting and tail biting [11]. Similarly, overcrowding of domestic chickens has been reported to lead to severe feather-pecking behavior, resulting in death, indicating that the survival probability of an individual depends on the genotype of its caged companions [12]. More evidence suggests that the role of SGEs in the process of evolution and domestic breeding cannot be ignored [13,14,15,16].

In current pig production systems, pigs are housed in groups, and social interaction-affected traits influence the phenotypes of group mates, such as behavior [17], growth rate [18], body weight [19,20], feed intake [21], and average daily gain (ADG) [22]. Previous studies have demonstrated the genetic parameters of SGEs [23,24] and the genetic correlation between DGEs and SGEs in carcass and growth performance [25,26]. The genome-wide association study (GWAS) approach has been employed to reveal key loci for DGEs and SGEs on socially affected traits in pigs [27,28,29]. However, traditional breeding programs for domestic pigs have relied on classical methods, such as selection based on estimated breeding values. These methods primarily account for an individual’s DGEs while neglecting the SGEs of other individuals [8]. Consequently, the use of classical selection methods for traits influenced by social interactions has sometimes resulted in unpredictable outcomes, including adverse effects like tail biting in pigs, despite significant improvements in growth performance [30,31]. Camerlink et al. assessed aggression in pigs that were divergently selected for SGEs on growth. They found no significant difference in aggression between the next-generation pigs selected for low or high SGEs [17]. Although SGEs are prevalent in various traits of domesticated animals, the mechanisms and pathways associated with these effects remain unclear.

Our previous study estimated the DGEs and SGEs for six socially affected traits, revealing a high correlation between DGEs and SGEs for RFI compared to the other traits [32]. We found that SGEs significantly affect the RFI in pigs. However, the intrinsic genetic characteristics and regulatory mechanisms of SGEs influencing the RFI from one individual to another remain unknown. This study aims to identify key candidate genes, pathways, and fundamental mechanisms related to SGEs on the RFI by examining the differences in the mRNA and protein expression in the liver and ileal mucosa of Duroc pigs with extremely low or high SGEs.

## 2. Methods

### 2.1. Ethical Approval

All the experimental procedures involving animals were approved by the Institutional Review Board and the Institutional Animal Care and Use Committee of the Sichuan Agricultural University under permit number No. 2020202051. All the methods in this study were performed in accordance with the institutional ethical standards in compliance with the ARRIVE guidelines (https://arriveguidelines.org, accessed on 4 October 2022) and all other relevant guidelines and regulations.

### 2.2. Animals

This study utilized 209 Duroc gilts from the breeding farm operated by Sichuan New Hope Liuhe Pig Breeding Technology Co., Ltd. (Chengdu, China). The exclusive use of gilts in this study minimized confounding effects from sex-based physiological differences, hormonal influences, and varied social behaviors, allowing for a clearer analysis of the feeding behavior, residual feed intake, and associated molecular mechanisms. The pigs were randomly assigned to 20 pens, with 10 to 12 pigs per pen. Each pen was equipped with feed intake recording equipment (Osborne, KS, USA), which tracked individual feeding behavior using the unique electronic identification tag on each pig’s ear. The system recorded the start time of feeding, feeding duration (i.e., time spent at the feeder per visit), feed consumption, and body weight at each visit to the feeder. The pigs had an average initial age of 93 days, with an average weight of 33.6 kg. The recording period lasted 12 weeks, with the pigs reaching an average age of 177 days and an average weight of 111 kg. All the pigs were fed a commercial corn–soybean diet formulated according to body weight, in compliance with the Chinese standard GB/T 5915-2020 [33], without antibiotics or drugs. Clean water was provided ad libitum, and a veterinarian regularly checked their health condition throughout the experiment.

### 2.3. Data Collection and Social Status Evaluation

Firstly, we conducted quality control on the recorded feeding data for each pig. We retained the data for daily feed intake ranging from 0.5 kg to 4.5 kg, daily feeding frequency of 2 to 20 times, and daily feeding duration of 5 min to 2 h. Subsequently, we calculated the initial body weight (W1), final body weight (W2), total time spent at the feeder per day (TPD), total feed intake (TFI), number of visits to the feeder per day (NVD), average feed intake per visit(AFI), average daily feed intake (ADFI), and average daily gain (ADG) for each pig. The backfat thickness between the 6th and 7th ribs was measured using real-time B-mode ultrasound (MyLab™X7, ESAOTE, Genova, Italy).

The displacement success (DS) percentage was calculated using the method outlined by Kranendonk et al. [34]. The pigs were equipped with individual electronic tags and monitored using the electronic feeding system, which automatically recorded each visit to the feeder, including the pig’s identification number, entry time, and exit time. Displacement events were inferred when one pig entered the feeding station within 2 s of another pig’s departure. The percentage of displacement success was determined as follows (the higher the displacement success rate, the higher the social status): [the number of times succeeding another gilt within 2 s ÷ (the number of times succeeded by another gilt within 2 s + the number of times succeeding another gilt within 2 s)] × 100.

#### RFI and SGE Calculation

The calculation of the RFI was based on the following formula [35]:

(1) RFI=ADFI−1.41ADG−2.83BF−110.9AMW

where ADFI = TFI/test days, ADG = (W2 − W1)/test days. The average metabolic body weight (AMW) for each individual was calculated using the following formula [36]:

(2) $$AMW=\frac{\left({W2}^{1.6}-{W1}^{1.6}\right)}{\left[1.6\times \left(W2-W1\right)\right]}$$

Subsequently, we used the DMU software (version 6) [37] to calculate the direct genetic effects and social genetic effects of the RFI. The following full model was built for the RFI trait [7].

(3) $$\;Y_{RFI}=\;Xb\;+\;Z_{d}a_{d}\;+Z_{s}a_{s}\;+\;Wl\;+\;Vg\;+\;e$$

where $Y_{RFI}$ is the phenotypic value vector of the RFI; b is the vector of the fixed effects, including the tested year and month; $a_{d}$ is a vector of a random DGE; $a_{s}$ is a vector of a random SGE; l is a vector of random litter effects, with $l\sim N(0,I_{l}\sigma_{l}^{2})$; g is a vector of random group (pen) effects, with $g\sim N(0,I_{g}\sigma_{g}^{2})$; e is a random residual vector, with $e\sim N(0,I_{e}\sigma_{e}^{2})$; and X, $Z_{d}$, $Z_{s}$, W and V are the incidence matrix of b, $a_{d}$, $a_{s}$, l and g, respectively. $I_{e}$, $I_{l}$ and $I_{g}$ are identity matrices. The additive genetic relationship matrix (A) used in the model was constructed from pedigree information. The variance–covariance matrix of $a_{d}$ and $a_{s}$ is denoted as:

(4) σAd2σAdsσAdsσAs2⊗A

where A is the additive genetic correlation matrix; and $\sigma_{A_{d}}^{2}$, $\sigma_{A_{s}}^{2}$ and $\sigma_{A_{ds}}$ are the genetic variance and covariance between the direct effects and social effects. At the ith row of $Z_{s}$, the group members of individual *i* were set to 1 and the others were set to 0. The group was defined based on the pen allocation, as pigs in the same pen were assumed to interact socially.

### 2.4. Sample Collection

The HPD95% interval of an SGE was calculated based on the estimated $\sigma_{A_{s}}^{2}$(−${1.96\sigma }_{A_{s}}/\sqrt{n}$ < M < ${1.96\sigma }_{A_{s}}/\sqrt{n}$) and assumed a normal distribution of the posterior SGE estimates. Animals with SGE values outside of this interval were classified as extreme. From these, four pigs with the highest SGEs (HS) and four with the lowest SGEs (LS) were selected from these extreme animals. These pigs were not explicitly balanced for pen or other factors, which may have introduced residual confounding. The pigs were slaughtered in accordance with the Live Pig Slaughter Guidelines (GB/T 17236-2019) [38], approved by the General Administration of Quality Supervision, Inspection, and Quarantine of the People’s Republic of China and the Standardization Administration of the People’s Republic of China. The pigs were starved overnight but allowed unlimited access to water before being slaughtered the next day. They were stunned with carbon dioxide prior to slaughter. The ileal mucosa and liver were aseptically separated and transferred into sterile 15 mL cryovials immediately before being frozen in liquid nitrogen for storage. To prevent cross-contamination, separate utensils were used for each sample.

### 2.5. RNA Sequencing and Quantification of Expression Levels

Approximately 0.1 g of liver and ileal mucosa samples were collected for RNA extraction. RNA was extracted according to the TRIzol reagent extraction instructions. The quality of total RNA was detected by RNA-specific agarose electrophoresis and Agilent 2100. RNA samples with RIN values greater than seven were considered qualified. Qualified RNA was sequenced by strand-specific library construction in BGI Company (Shenzhen, China), and the sequencing platform was DNBSEQ and BGISEQ-500.

FastQC was used for quality control of the raw data and filtered using SOAPnuke. Clean reads were then mapped to the reference genome Sscrofa 11.1 using Hisat2. Differential expression analysis was then performed using DESeq2 to identify differentially expressed RNAs. Differentially expressed genes (DEGs) were selected based on a false discovery rate (FDR) < 0.05. This threshold ensures biologically meaningful expression differences while controlling for multiple testing. Hierarchical clustering analysis was performed to examine the overall expression patterns of the DEGs.

### 2.6. iTRAQ-Based Quantitative Proteomic Analysis

Approximately 50 μg of liver and ileum mucosa tissue was processed for protein extraction. The tissue was ground, lysed, centrifuged, and precipitated to obtain protein solutions. The protein concentrations were then determined using the Bradford assay kit. Afterward, approximately 100 μg of the sample was taken for labeling according to the instructions of the AB assay kit. The labeled samples were subjected to liquid-phase separation using the Shimadzu LC-20AD liquid phase system. After liquid phase separation, the peptides were ionized by a nanoESI source and entered into a tandem mass spectrometer Q-Exactive HF X (Thermo Fisher Scientific, San Jose, CA, USA) for data-dependent acquisition (DDA) mode detection. The main parameters were as follows: ion source voltage was set to 1.9 kV; primary mass spectrometry scan range 350~1500 m/z; the resolution was set to 60,000; secondary mass spectrometry starting m/z was fixed at 100; the resolution was 15,000. The parent ions for the secondary fragmentation were selected as the top 20 parent ions with charge 2+ to 6+ and peak intensity over 10,000. The ion fragmentation mode used was HCD and fragment ions were detected in Orbitrap. The dynamic exclusion time was set to 30 s and the AGC was set to 3E6 for primary and 1E5 for secondary.

The MS/MS data were converted into MGF format and compared to the NCBI porcine genus (Sus) database using the protein identification software MASCOT2.3.02 (Matrix Science, London, UK). The iTRAQ data were quantified using IQuant v2.0.1 software, which was first filtered with 1% FDR at the spectrum/peptide level to obtain a list of significantly identified spectra and peptides. The peptides were then used for protein assembly and a series of proteomes were generated. An additional filtering step was applied at the protein level using an FDR threshold of 1% to control the false positive rate of protein identification. The strategy employed was to pick proteins based on their FDR values. Hierarchical clustering analysis was performed to examine the differential protein patterns between samples and groups.

### 2.7. Weighted Gene Co-Expression Network Analysis

Weighted gene co-expression network analysis (WGCNA) was performed to identify the modules of co-expressed genes associated with the RFI. Genes with fragments per kilobase million (FPKM) values less than 1 were excluded, and the remaining genes were used for the analysis. The analyses were performed separately for the HS and LS groups using the same parameters. The gene expression matrices were checked for outliers using the goodSamplesGenes function to ensure data quality, and no samples or genes were removed as outliers. Using the pickSoftThreshold function, soft-threshold powers of 18 (liver) and 16 (ileum) were selected to approximate the scale-free topology. A topological overlap matrix (TOM) was computed to measure the network interconnectedness, and hierarchical clustering based on the TOM dissimilarity was performed. The gene modules were identified using a dynamic tree cut method with the parameters set to minModuleSize = 30, deepSplit = 2, and pamRespectsDendro = FALSE. The modules were then merged based on the eigengene similarity using a threshold cut height of 0.25. The relationships between the module eigengenes and the RFI were assessed using the Pearson correlation, and the module–trait relationships were visualized through heatmaps. Modules showing strong or significant correlations with the RFI were selected for the subsequent functional enrichment analysis to explore the associated biological processes and pathways. All the WGCNA analyses were performed in R (version 4.4.1) using the WGCNA package (version 4.3.3) [39].

### 2.8. Analysis of DEG, DEP, and Module Enrichment Pathways

The enrichment analysis was performed using DAVID Bioinformatics Resources 6.8 (https://david.ncifcrf.gov/, accessed on 20 April 2025) to identify potential mechanisms and pathways. The analysis was conducted within the context of the Kyoto Encyclopedia of Genes and Genomes (KEGG) pathway [40] and Gene Ontology (GO) terms, including the biological process (BP), cellular component (CC), and molecular function (MF). Statistical significance was set at *p*-value < 0.05 and gene count ≥ 2.0.

### 2.9. Validation of RT-qPCR

Real-time fluorescence quantitative PCR was employed to validate the RNA sequencing results. The genes selected for validation were randomly chosen from the RNA sequencing data. The quantitative primer design was performed using Primer 5.0 and synthesized by Shanghai Biotech Co., Ltd. (Shanghai, China). The total RNA was isolated using the TRIzol reagent (OMEGA, Norcross, GA, USA; Genstar, Beijing, China) and reverse transcribed using the one-step gDNA Removal and cDNA Synthesis SuperMix kit according to the manufacturer’s instructions. The qPCR of all the genes was performed on a 7500 Real-Time PCR system (Applied Biosystems, Warrington, UK) using the fluorescent quantitative reagent kit (Biomiga, San Diego, CA, USA). Each sample was tested in triplicate. The Ct values for each gene were calculated using the 2^−△△CT^ method. To compare the qPCR results with the sequencing-based results, the average 2^−△△CT^ value for each gene was converted to fold change.

### 2.10. Statistical Analysis

The production data and feeding information were analyzed using a two-tailed Student’s *t*-test in the Statistical Package for Social Science program (SPSS 22.0, Chicago, IL, USA). A *p*-value < 0.05 was considered statistically significant. For all the differential expression mRNAs and proteins, we report the nominal *p*-values, adjusted for multiple testing using the modified Benjamini–Hochberg method to control the false discovery rate (FDR) [41]. Differentially expressed mRNAs were defined as those with an FDR < 0.05 and |log_2_FC| > 1, while differentially expressed proteins (DEPs) were defined as those with an FDR < 0.05 and |log_2_FC| > 1.2.

## 3. Results

### 3.1. Variance Components of Residual Feed Intake from a Social Genetic Model

We calculated the direct genetic effects (DGEs) and social genetic effects (SGEs) of the residual feed intake (RFI) for all 209 pigs. The variance and covariance between the direct and social genetic effects of the RFI were estimated and are presented in Table 1. The HPD95% interval for SGE was determined as −4.304 to 4.304. Based on this interval, we selected the extreme animals that were higher than 4.304 (HS) or lower than −4.304 (LS). Detailed information about the HS and LS is provided in Supplementary File S1.

### 3.2. Comparative Analysis of Feeding Behavior and Growth Traits in Pigs with Extreme Social Genetic Effects

The feeding behavior and productive performance of the selected pigs were analyzed and are presented in Table 2. As expected, there were significant differences in the residual feed intake (RFI), feed conversion ratio (FCR), and average daily weight gain (ADG) between the HS and LS groups (*p* < 0.001); the RFI, FCR, and ADG were significantly higher in the LS group. We further analyzed the four feeding-related behaviors with different SGEs, including the average daily feed intake (ADFI), total time spent at feeder per day (TPD), average feed intake per visit (AFI), and number of visits to the feeder per day (NVD). Consistent with the findings on the ADG, the HS group exhibited a lower ADFI compared to the LS group. The pigs in the HS group visited the feeder less frequently, yet they consumed more feed per visit. Notably, the TPD of the HS group was higher than that of the LS group, indicating that these pigs spent more extended periods at the feeder during each visit, enabling them to eat at a leisurely pace and fulfill their satiety. This phenomenon may be attributed to individuals with a high SGE possessing a higher social status, which enables them to feed in a more relaxed environment and spend more time at the feeder. Subsequently, we calculated the percentage of displacement success (DS) for each individual as a measure of their social status within the group (Table 2). The results indicated that the HS group had a higher social status than the LS group, suggesting that individuals in the LS group experience comparatively lower social standing within the group. Consequently, they may frequently encounter disturbances during feeding, thereby being compelled to visit the feeder more often in order to secure adequate food intake.

### 3.3. Differences in Transcriptome Profiles with Different Social Genetic Effects

The SGE of the RFI is related to variations in growth performance and feeding behavior, suggesting potential differences in gene expression. Considering that the main biological processes influencing the RFI are digestion and absorption, we selected the liver and ileum for transcriptome sequencing. The processed clean data were used for the subsequent analysis (Supplementary File S2).

### 3.4. Differential Gene Expression in the Liver Is Mainly Related to Mitochondria Functions and Oxidative Phosphorylation

**Figure 1:**
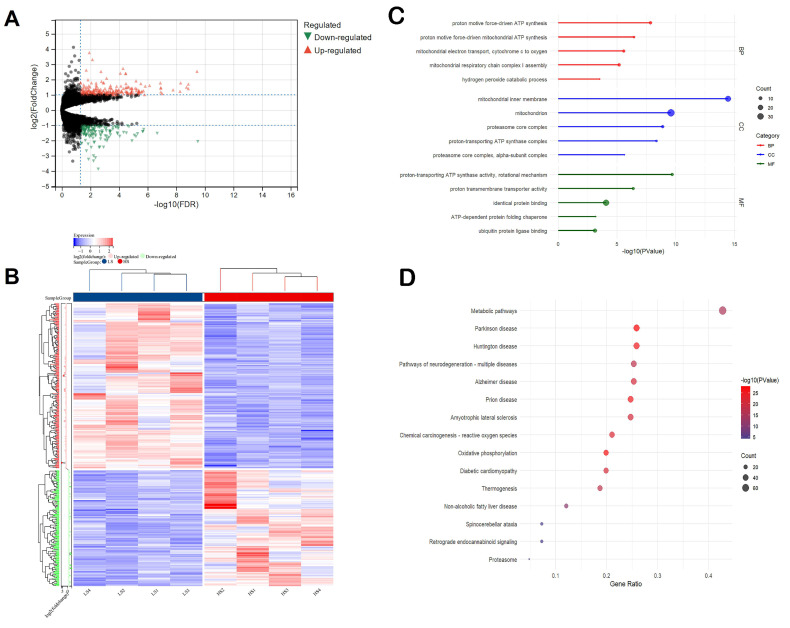
Significant differentially expressed mRNA in the liver tissue between the HS and LS groups. (**A**) Volcano plot of the differentially expressed mRNAs; upward-pointing red triangles represent upregulated mRNAs, downward-pointing green triangles represent downregulated mRNAs, and black dots represent mRNAs that are not significantly differentially expressed, (**B**) heatmap of the differentially expressed mRNA under clustering conditions, (**C**) top 5 GO terms: biological process (BP), cellular component (CC), molecular function (MF), and (**D**) analysis of the top 15 KEGG-enriched pathways of the DEGs.

Transcriptome expression analysis of the liver revealed 360 differentially expressed genes (DEGs) (Figure 1A). The HS group had 262 up-regulated and 98 down-regulated significant DEGs compared to the LS group (criterion: |log_2_FC| > 1, FDR < 0.05). Hierarchical clustering analysis of the DEGs indicated that our grouping results are relatively ideal (Figure 1B). *TCN1* and *CA3* were the most up- (log_2_FC = 5.48) and down-regulated (log_2_FC = −3.88) genes, respectively (Table 3).

To further investigate the functions of the DEGs, functional annotation was performed. The up-regulated DEGs were significantly involved in GO terms associated with proton motive force-driven ATP synthesis, proton motive force-driven mitochondrial ATP synthesis, and other mitochondrial functions, including electron transport, respiratory chain complex I assembly, and mitochondrial inner membrane (Figure 1C). The down-regulated DEGs were significantly associated with transmembrane transport, peripheral nervous system neuron development, and regulation of membrane potential (Supplementary File S4). We also performed a KEGG enrichment analysis of the DEGs to identify the central pathways. The up-regulated DEGs were significantly enriched in biological pathways associated with metabolic pathways, oxidative phosphorylation, thermogenesis, and pathways associated with other diseases (Figure 1D). The down-regulated DEGs were enriched in nitrogen metabolism, cell adhesion molecules, and metabolic pathways.

### 3.5. Differential Gene Expression in the Ileum Is Mainly Related to Cholesterol Metabolism, Fat Digestion and Absorption, and Amino Acid Biosynthesis

**Figure 2:**
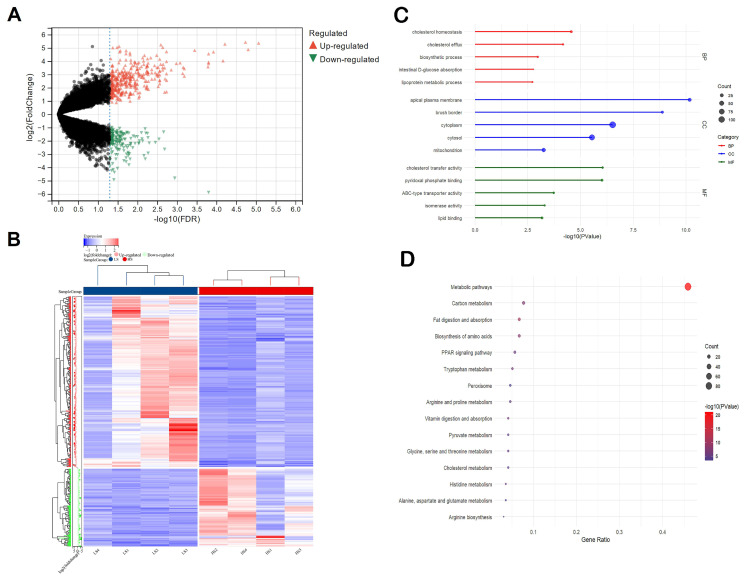
Significant differentially expressed mRNA in the ileum between the HS and LS groups. (**A**) Volcano plot of the differentially expressed mRNAs; upward-pointing red triangles represent upregulated mRNAs, downward-pointing green triangles represent downregulated mRNAs, and black dots represent mRNAs that are not significantly differentially expressed, (**B**) heatmap of the differentially expressed mRNA under clustering conditions, (**C**) top 5 GO terms: biological process (BP), cellular component (CC), molecular function, and (**D**) analysis of the KEGG pathway enrichment of the DEGs.

We also identified 546 significant DEGs (375 up-regulated genes and 171 down-regulated genes) in the ileum (criterion: |log_2_FC| > 1, FDR < 0.05) (Figure 2A). *RTL4* was the most up-regulated gene (log_2_FC = 6.09), while *GRM8* was the most down-regulated gene (log_2_FC = −3.82) in the HS group (Table 4). The hierarchical clustering heatmap analysis of the DEGs is presented in Figure 2B. Compared to the liver, the ileum exhibited a greater number of DEGs with larger fold changes. Furthermore, the gene expression patterns within each group were not entirely consistent.

The results of the GO enrichment analysis of the up-regulated DEGs showed that the most significant GO terms were related to cholesterol homeostasis, cholesterol efflux, biosynthetic process, intestinal D-glucose absorption, and lipoprotein metabolic process (Figure 2C). The down-regulated DEGs were associated with terms such as DNA integration, sodium ion transmembrane transport, positive regulation of transcription by RNA polymerase II, and amino acid transport. The KEGG pathway enrichment analysis revealed significantly enriched biological pathways for the up-regulated DEGs. These enriched pathways predominantly involve metabolic pathways, carbon metabolism, fat digestion and absorption, biosynthesis of amino acids, and PPAR signaling pathway (Figure 2D). The down-regulated DEGs were associated with KEGG pathways involving other types of O-glycan biosynthesis, Fc gamma R-mediated phagocytosis, Th17 cell differentiation, and glutamatergic synapse (Supplementary File S4).

To validate the reliability of the RNA-seq results, we randomly selected six genes from the commonly enriched differentially expressed genes. The results demonstrated a strong consistency between the RT-qPCR and RNA-seq data, indicating the reliability and high reproducibility of the RNA-seq results (Supplementary File S2).

### 3.6. Co-Expression Modules in the Liver Are Associated with Immune Regulation, Cholesterol Metabolism, and Mitochondrial Function

Weighted gene co-expression network analysis (WGCNA) was performed to identify the modules associated with the RFI of the SGE groups. After quality control, that is, removing genes with low expression levels, 8752 (HS group) and 9751 (LS group) genes from the liver were retained for network construction. Eight co-expression modules were identified in the HS group, with the number of genes per module ranging from 43 to 3955. In the LS group, six modules were detected, containing between 71 and 5406 genes (Supplementary File S5).

**Figure 3:**
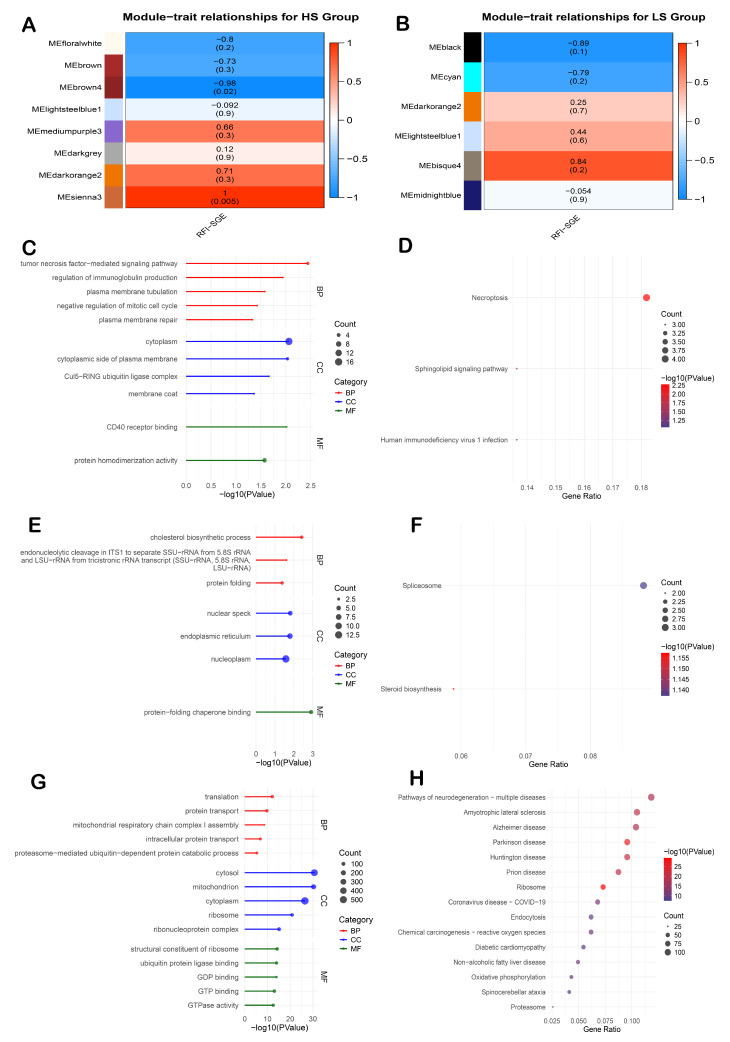
Moduletrait correlations and functional enrichment analysis of key co-expression modules identified by WGCNA in the liver tissue. (**A**) Module–trait correlation for the HS group, (**B**) module–trait correlation for the LS group, (**C**) top 5 GO terms: biological process (BP), cellular component (CC), molecular function of brown4 module, (**D**) KEGG pathways enrichment of the brown4 module, (**E**) top 5 GO terms: biological process (BP), cellular component (CC), molecular function of sienna3 module, (**F**) KEGG pathways enrichment of the sienna3 module, (**G**) top 5 GO terms: biological process (BP), cellular component (CC), molecular function of black module, and (**H**) KEGG pathways enrichment of the black module.

For the HS group, the brown4 and sienna3 modules were identified as the top modules associated with the RFI (Figure 3A). The brown4 module, consisting of 43 genes, was negatively correlated with the RFI (r = −0.98, *p* < 0.05), while the sienna3 module, comprising 64 genes, was positively correlated with the RFI (r = 0.99, *p* < 0.05). In the LS group, the black module (Figure 3B), containing 2057 genes, showed a negative correlation (r = −0.89, *p* = 0.1).

Functional enrichment analysis revealed biological processes associated with the top modules. In the HS group, genes in the brown4 module were significantly enriched in GO terms related to the regulation of immunoglobulin production and plasma membrane processes (Figure 3C). Genes within the sienna3 module were primarily involved in cholesterol biosynthetic processes and protein folding (Figure 3E). In the LS group, black module genes were associated with translation, protein transport, and mitochondrial function (Figure 3G).

Pathway analysis further demonstrated the enrichment of specific KEGG pathways. In the HS group, the brown4 module genes were significantly enriched in the sphingolipid signaling pathway (Figure 3D), while genes in the sienna3 module were enriched in the spliceosome and steroid biosynthesis pathways (Figure 3F). In the LS group, black module genes were enriched in oxidative phosphorylation and various disease-related pathways (Figure 3H).

### 3.7. Co-Expression Modules in the Ileum Are Associated with Fatty Acid Metabolism and Protein Degradation Pathways

From the ileum transcriptome data, 10,658 genes (HS group) and 9634 genes (LS group) were retained for the WGCNA analysis. Eight co-expression modules were identified in the HS group, with module sizes ranging from 372 to 3114 genes. In the LS group, seven modules were identified, with module sizes ranging between 69 and 4618 genes (Supplementary File S5).

**Figure 4:**
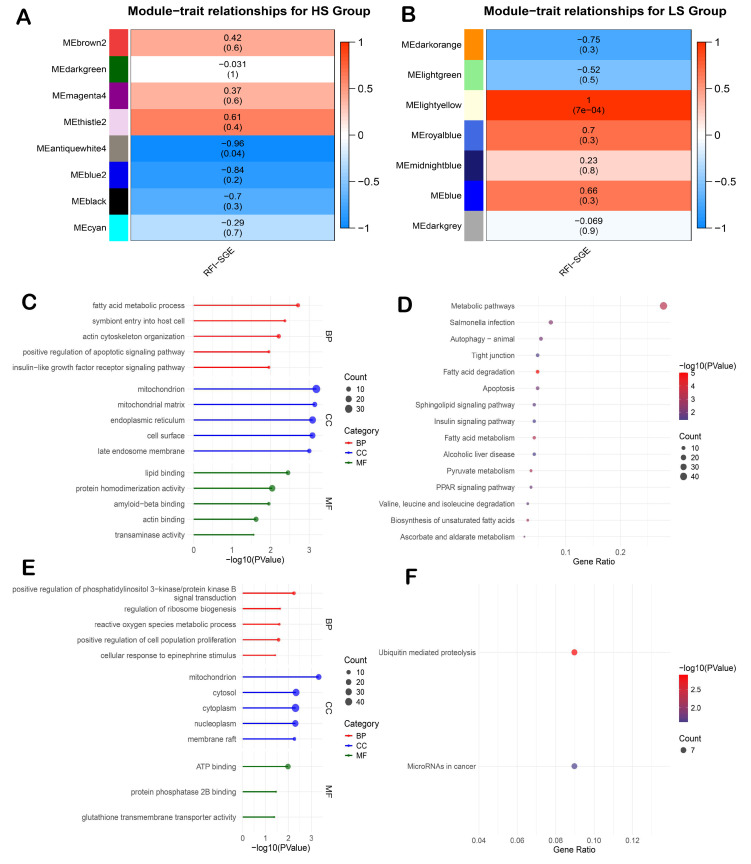
Module–trait correlations and functional enrichment analysis of key co-expression modules identified by WGCNA in the ileum tissue. (**A**) Module–trait correlation for the HS group, (**B**) module–trait correlation for the LS group, (**C**) top 5 GO terms: biological process (BP), cellular component (CC), molecular function of antiquewhite4 module, (**D**) KEGG pathways enrichment of the antiquewhite4 module, (**E**) top 5 GO terms: biological process (BP), cellular component (CC), molecular function of the lightyellow module, and (**F**) KEGG pathways enrichment of the lightyellow module.

In the HS group, the antiquewhite4 module was identified as the top module (Figure 4A), showing a strong negative correlation with the RFI (r = −0.96, *p* < 0.05). In the LS group, the lightyellow module was identified as the top module (Figure 4B) and was positively correlated with the RFI (r = 0.99, *p* < 0.05).

Functional enrichment analysis showed that the genes in the antiquewhite4 module (HS group) were significantly involved in fatty acid metabolic processes and other mitochondrial-related functions (Figure 4C). In the LS group, the genes in the lightyellow module were enriched in processes related to the regulation of ribosome biogenesis and reactive oxygen species metabolism (Figure 4E).

KEGG pathway analysis further indicated that the antiquewhite4 module in the HS group was significantly enriched in pathways including metabolic pathways, fatty acid degradation, and fatty acid metabolism (Figure 4D). In the LS group, the lightyellow module genes were enriched in the ubiquitin-mediated proteolysis pathway (Figure 4F).

### 3.8. Proteomic Analysis Revealed Differentially Expressed Proteins

Based on the transcriptome sequencing, we identified differentially expressed genes in the liver and ileum, which may serve as the molecular basis for the SGEs on the RFI. To further screen for core genes, we performed proteomic analysis of the liver and ileum using iTRAQ technology. We aimed to identify functional proteins that are associated with the SGEs on the RFI and performed a combined analysis with the transcriptome data.

**Figure 5:**
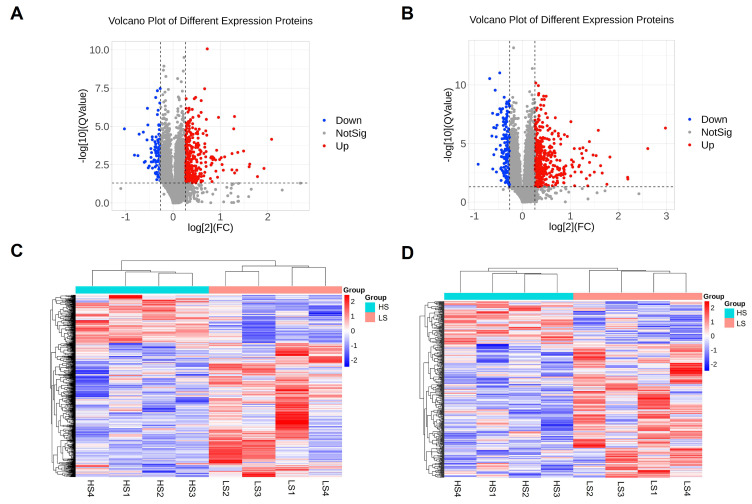
Quantitative proteome analysis of differentially expressed proteins in different tissues using iTRAQ. (**A**) Volcano plot of the log_2_-fold changes in protein abundance in the liver and their statistical significance, (**B**) volcano plot of the log_2_-fold changes in protein abundance in the ileum and its statistical significance, (**C**) differential protein map in the liver, and (**D**) differential protein map in the ileum.

We identified 6424 proteins in the liver and 7866 proteins in the ileum, with 4716 co-expressed proteins. In the liver, 606 differentially expressed proteins (DEPs) were found, comprising 446 up-regulated and 160 down-regulated proteins in the LS group (Figure 5A). In the ileum, there were 396 DEPs, of which 302 were up-regulated and 94 were down-regulated (|log_2_FC| > 1.2, FDR < 0.05) (Figure 5B). The hierarchical clustering analysis indicated that the two groups showed distinct patterns of protein changes, with most samples within each group consistently showing up-regulation or down-regulation, suggesting good reproducibility (Figure 5C,D).

**Figure 6:**
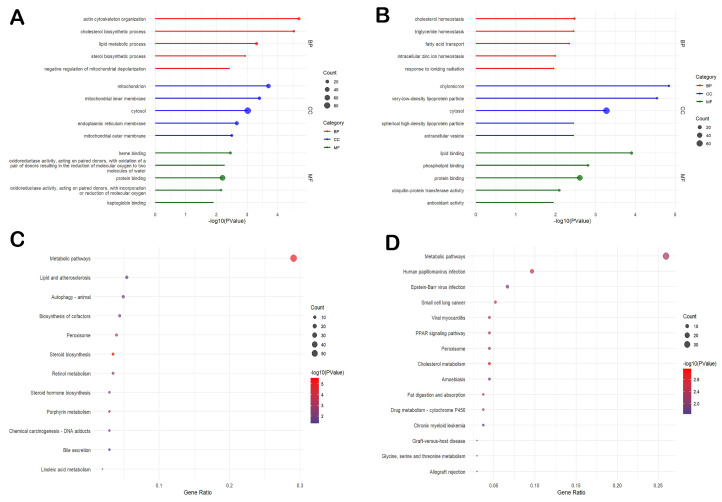
Functional enrichment analysis of differentially expressed proteomes in the liver and ileum. (**A**) GO enrichment analysis of the DEPs in the liver: biological process (BP), cellular component (CC), molecular function (MF), (**B**) GO enrichment analysis of the DEPs in the ileum: biological process (BP), cellular component (CC), molecular function (MF), (**C**) KEGG pathway enrichment analysis of the DEPs in the liver, and (**D**) KEGG pathway enrichment analysis of the DEPs in the ileum.

To further investigate the differential proteins involved in the SGEs on the RFI in pigs and to unravel the underlying molecular mechanisms, we performed GO annotation and KEGG enrichment pathway analysis on the DEPs in each comparison group. In the liver, the up-regulated DEPs were significantly enriched in biological processes such as actin cytoskeleton organization, cholesterol biosynthetic process, lipid metabolic process, and sterol biosynthetic process, as well as cellular components such as mitochondrion, mitochondrial inner membrane, and others (Figure 6A). The down-regulated DEPs in the liver were associated with GO terms such as positive regulation of mRNA splicing, via spliceosome, regulation of translation, and extracellular matrix disassembly. In the ileum, the up-regulated DEPs were significantly enriched in biological processes such as cholesterol homeostasis, triglyceride homeostasis, and fatty acid transport, while the cellular structures included chylomicron, very low-density lipoprotein particle, and cytosol (Figure 6B). The down-regulated DEPs in the ileum were involved in processes such as positive regulation of canonical NF-kappaB signal transduction, high-density lipoprotein particle assembly, phospholipid efflux, and others (Supplementary File S4).

We also performed a KEGG pathway enrichment analysis of the DEPs from both tissues. The pathways involved in the up-regulated DEPs in the liver include metabolic pathways, lipid and atherosclerosis, biosynthesis of cofactors, peroxisome, and steroid biosynthesis (Figure 6C). The down-regulated DEPs were associated with metabolic pathways, spliceosome, RNA degradation, and biosynthesis of amino acids. In the ileum, the up-regulated DEPs enrichment primarily occurred in metabolic pathways, fat digestion and absorption, cholesterol and various amino acid metabolism, the PPAR signaling pathway, and pathways related to viral infection and cancer (Figure 6D). The down-regulated DEPs in the ileum were associated with pathways related to ABC transporters.

### 3.9. Protein–Protein Interaction Network Analysis and Hub Protein Selection

**Figure 7:**
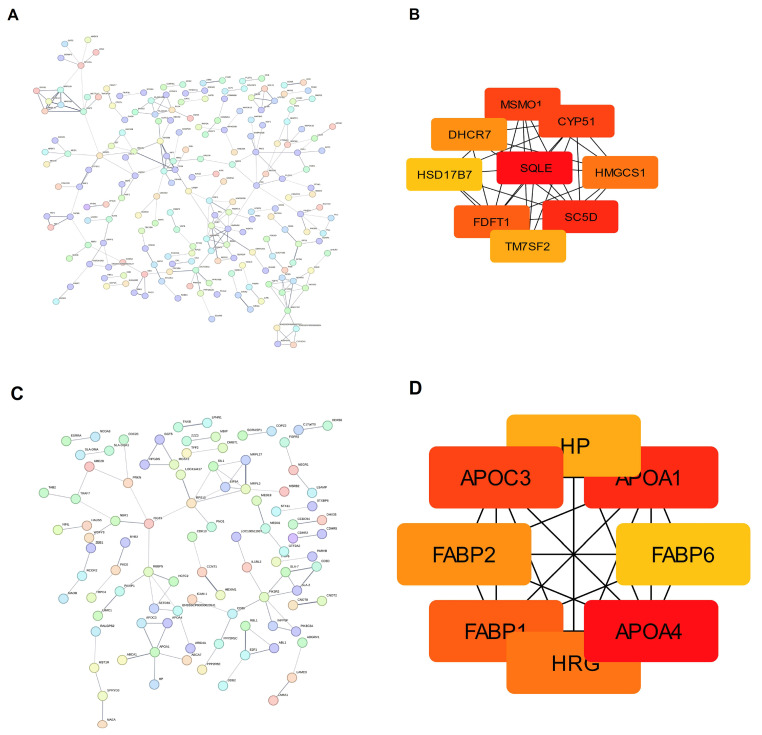
The differential proteins screened from the two tissues were subjected to protein–protein interaction network analysis. (**A**) PPI network of the DEPs in the liver, (**B**) the core gene of the PPI network in the liver, (**C**) PPI network of the DEPs in the ileum, and (**D**) the core gene of the PPI network in the ileum.

The DEPs in the liver and ileum were separately introduced into the STRING database to obtain the functional protein association networks. The protein–protein interaction (PPI) network in the liver tissue was constructed with 533 nodes and 206 edges, with a high confidence score of 0.400 and an enriched *p*-value of 3.29 × 10^−7^ (Figure 7A). Nine hub genes were selected using the cytoHubba plugin: *SQLE*, *SC5D*, *HMGCS1*, *DHCR7*, *CYP51*, *MSMO1*, *HSD17B7*, *FDFT1*, and *TM7SF2* (Figure 7B). Our functional analysis of the core gene found that the core gene in the liver was involved in the cholesterol biosynthetic process and steroid biosynthesis. The PPI network in the ileum consisted of 334 nodes and 312 edges, with a confidence score of 0.400 and an enrichment *p*-value of 0.00236 (Figure 7C). Eight hub genes were selected using the cytoHubba plugin: *APOA1*, *APOA4*, *APOC3*, *FABP1*, *FABP2*, *FABP6*, *HRG*, and *HP* (Figure 7D). The KEGG enrichment pathways of these hub genes were mainly fat digestion and absorption, PPAR signaling pathway, and cholesterol metabolism (Supplementary File S4).

### 3.10. Association Analysis of the Differentially Expressed Genes and Proteins

**Figure 8:**
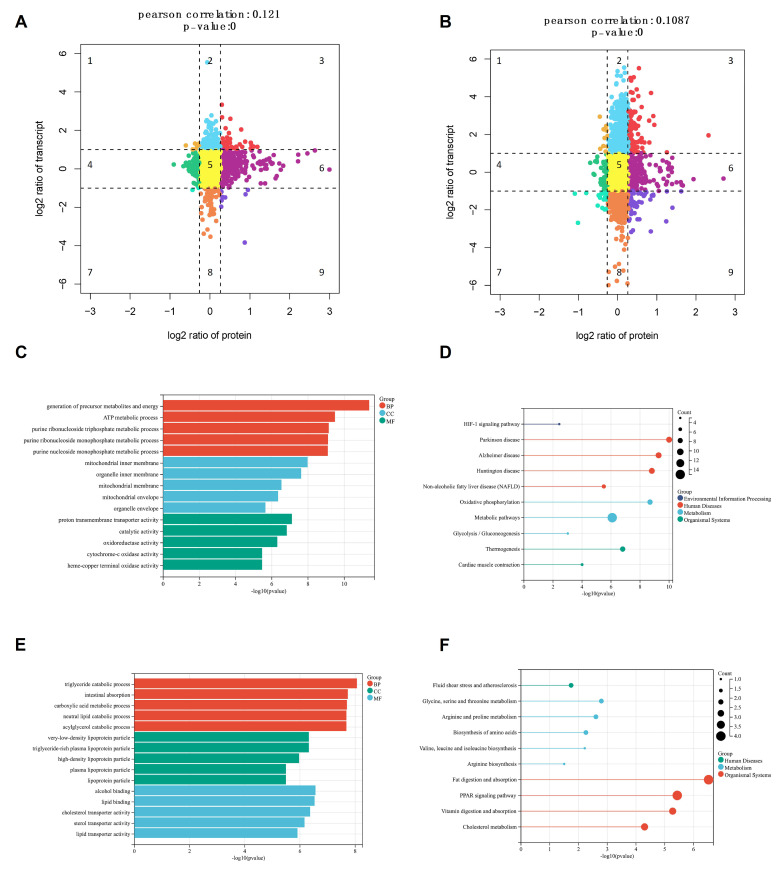
The differential proteins screened from the two tissues were subjected to PPI interaction network analysis. (**A**) Nine-quadrant chart of the mRNA expression ratios and protein expression ratios in the liver; different colors denote different quadrants, representing various patterns of expression between mRNA and protein levels, (**B**) nine-quadrant chart of the mRNA expression ratios and protein expression ratios in the ileum; different colors denote different quadrants, representing various patterns of expression between mRNA and protein levels, (**C**) GO analysis of the DEG-DEPs in the liver tissue: biological process (BP), cellular component (CC), molecular function (MF), (**D**) KEGG pathways co-enriched by the DEG-DEPs in the liver tissue, (**E**) GO analysis of the DEG-DEPs in the ileum: biological process (BP), cellular component (CC), molecular function (MF), and (**F**) KEGG pathways co-enriched by the DEG-DEP in the ileum.

To further identify critical genes related to the SGEs, the combined transcriptome and proteome data were analyzed. First, the co-expressed proteins in the two omics were screened, and a nine-quadrant map was drawn according to the fold difference between the mRNA and the protein. Quadrants 1, 2, and 4 indicate that the protein abundance is lower than the RNA abundance; in quadrants 3 and 7, the RNA corresponds to related proteins; quadrant 5 shows the protein and RNA ubiquitous expression, with no difference; and quadrants 6, 8, and 9 show the protein abundance is higher than the RNA abundance (Figure 8A,B). Among them, 29 and 19 DEG-DEPs were co-upregulated in the liver and ileum, respectively.

We performed PPI network analysis of these 29 and 19 DEG-DEPs using the STRING network. Details of the DEG-DEPs are shown in Table 5. Analysis of their functions revealed that most of the differential genes in the liver were involved in various metabolic processes, mitochondrial functions, and oxidative phosphorylation (Figure 8C,D). The differential genes in the ileum were mainly involved in various catabolic processes, digestion and absorption of fat, cholesterol metabolism, glycine, serine, and threonine metabolism, and arginine and proline metabolism (Figure 8E,F).

Furthermore, the correlation coefficients between the mRNA and the protein in these two tissues were 0.121 and 0.1087, respectively. The majority of the differentially expressed proteins showed a low correlation with their corresponding transcript levels, indicating that post-transcriptional modifications might play a primary role in regulating the SGEs on the RFI in pigs.

## 4. Discussion

Social genetic effects (SGEs) are the heritable influence of one individual on the phenotype of its social partners. They are critical in livestock and aquaculture, where selecting for SGEs has been proposed to reduce harmful behaviors [42]. SGEs significantly affect feeding behavior and production traits. For example, low-SGE individuals often display competitive and aggressive behaviors, while high-SGE individuals exhibit cooperative traits [17]. Studies revealed a strong negative correlation between the SGEs and the direct genetic effects (DGEs) on the feeding station occupation time [21]. However, this study observed a low positive correlation (0.128 ± 0.11) between the SGEs and the DGEs on the residual feed intake (RFI), suggesting that the relationship between these effects may be trait-dependent. The positive correlation may indicate that pigs with favorable DGEs for the RFI may also exhibit beneficial SGEs for the same trait. The results from this study indicated that pigs with a lower RFI exhibit higher SGEs. However, classical animal models do not take into account the effects of group members on individuals and may lead to biased estimates of the DGEs on the RFI. Therefore, in the selection for the RFI, it is advisable to utilize animal models that incorporate SGEs in order to fully consider their impact on the phenotype.

In this study, we found that individuals with higher SGEs spent a longer time but had fewer visits to the feeder. They consumed a lesser overall amount of food while exhibiting a higher intake per visit. Similar feeding patterns and social behaviors were observed in other studies [43]. A previous study ranked 12 pigs within the same group and found that pigs with higher rankings visited the electronic feeding station less frequently but stayed for a longer time and consumed more feed. A study by Camerlink et al. observed that piglets from a line selected for high SGEs were slower to explore the feed upon weaning in a new environment, which may indicate that these pigs prioritized social assessment or hierarchy establishment over immediate feeding [20]. Among captive macaques, females with lower social status exhibit signs of chronic stress and consume more food than those with higher social status [44]. Therefore, we speculate that individuals with high SGEs have higher social status, allowing them to feed in a more relaxed environment. This enables them to occupy the feeder for longer periods while consuming more food without being disturbed. In contrast, individuals with low SGEs may experience frequent interruptions during feeding due to their lower social status, leading to a reduced intake per visit and increased feeder visits. However, other studies produced differing results. For instance, Herrera-Cáceres et al. [21] found that dominant animals exhibit a higher feeding frequency, while Nielsen et al. reported no relationship between feeding behavior and social class. They suggested that the correlations between performance and social behavior may have been masked by environmental factors such as the space allowance and straw provision. Alternatively, they proposed that aggression and growth might be independent traits [45,46]. These conflicting findings suggest that the relationship between feeding behavior and social status may be complex and may be influenced by various factors. Overall, evaluating the social status of pigs in group feeding remains challenging, requiring further exploration to elucidate the relationship between SGEs, social status, and feeding behavior.

This study analyzed the liver and ileum transcriptomes and proteomes of pigs with extremely high or low SGEs. Although the sample size was limited, which impaired the inclusion of potential confounders such as pen as covariates in the differential analysis, this study therefore provides preliminary insights into the biological mechanisms underlying SGEs and their potential links to feed efficiency. The differential gene and protein expressions observed in the liver potentially reflect physiological responses to feeding behaviors associated with the SGEs on the residual feed intake. In addition, the ileum’s role in nutrient absorption and neurotransmitter secretion, which potentially influence gut–brain signaling, may play a more direct role in the causation of SGE-mediated effects on the RFI. A comparison between the LS and HS groups revealed a significant number of differentially expressed genes in both the liver and ileum. In the liver, *TCN1* and *CA3* were the most up-regulated and down-regulated genes, respectively. *TCN1* encodes a vitamin-B12-binding protein essential for nutrient utilization. *TCN1* has been reported to be located near a quantitative trait locus for the RFI in cattle. Also, vitamin B12 supplementation has been linked to improved weight gain in calves [47] and improved average daily gain and feed conversion in pigs [48], suggesting *TCN1*-mediated B12 uptake could influence feed efficiency. *CA3* is involved in muscle metabolism and energy homeostasis. In pigs, the *CA3* expression in muscle has been reported to correlate positively with the intramuscular fat levels, indicating a role in muscle fuel utilization and lipid metabolism [49]. Differences in *CA3* expression or activity could reflect a shift in how muscle uses nutrients, thereby influencing an animal’s energy expenditure and feed efficiency. Furthermore, *RTL4* was the most up-regulated gene, while *GRM8* was the most down-regulated gene in the HS group in the ileum. In mice, *RTL4* encodes a secreted protein that responds to noradrenaline signaling. Knockout mice lacking *RTL4* show pronounced behavioral changes, such as increased impulsivity, impaired short-term memory, and poor adaptation to new environments, and it has been implicated as a causative gene in certain neurodevelopmental disorders in humans [50]. The behavioral phenotypes observed in *RTL4* knockout mice may suggest a potential role for this gene in modulating social and feeding behaviors, which could be relevant to the SGEs in pigs. *GRM8* is involved in appetite regulation and social feeding interactions. For instance, a polymorphism in *GRM8* (rs2237781) was associated with higher dietary restraint in humans, suggesting that glutamate signaling could influence how individuals regulate their eating behavior [51]. This potential role remains hypothetical and requires further investigation in pigs.

The functional enrichment results of the DEGs in both tissues were quite consistent, highlighting the functional differences. In the liver, the DEGs were primarily related to mitochondrial functions and oxidative phosphorylation. The ileum plays a critical role in nutrient absorption and gut microbiota regulation, both of which influence feed efficiency. The DEGs in the ileum were mainly associated with cholesterol metabolism and metabolic pathways related to fat digestion and absorption, as well as amino acid biosynthesis, suggesting that the SGEs may impact how efficiently nutrients are extracted from feed.

Weighted gene co-expression network analysis was performed on the liver and ileum transcriptomes to identify biologically meaningful modules associated with the RFI. In the liver of the HS group, the brown4 module was enriched in processes related to immunoglobulin production and plasma membrane regulation, while the sienna3 module was associated with cholesterol biosynthesis and protein folding. These findings suggest that immune regulation and cholesterol metabolism processes may be important contributors to the improved feed efficiency in the HS group. In the LS group, the black module was enriched in processes related to translation, protein transport, and mitochondrial function. In the ileum, the antiquewhite4 module in the HS group was associated with fatty acid metabolic mechanisms, while the lightyellow module in the LS group was enriched in ribosome biogenesis and reactive oxygen species metabolism. These results suggest that the nutrient metabolism mechanisms in the HS group and the stress response mechanisms in the LS group may contribute to the differences in feed intake efficiency under social interactions.

Proteomic analysis was performed to further explore the biological changes associated with the SGE groups. However, the number of differentially expressed proteins between the two groups was significantly lower across both tissues compared to the number of differentially expressed genes, leading to a lower correlation between the proteome and the transcriptome. This might be due to complex post-transcriptional regulation. Additionally, the enrichment analysis of the differentially expressed proteins predominantly highlighted biosynthetic processes and mitochondrial-related functions in the liver, as well as cholesterol metabolism, including cholesterol homeostasis, and related processes such as fatty acid transport, and fat digestion and absorption, in the ileum. These findings align with the functional enrichment results of the differentially expressed genes. Consequently, an integrated analysis of the transcriptome and proteome is essential to further identify key genes.

Mitochondria are double-membrane organelles primarily responsible for energy generation. In recent years, increasing evidence has shown a clear link between mitochondrial function and social behavior [52,53]. For example, in a rat model of autism, treatment with a ketogenic diet enhances mitochondrial function and ameliorates impaired mitochondrial respiration and deficits in social behavior [54]. The mitochondrial respiratory chain is located on the inner mitochondrial membrane, which consists of five complexes, namely complex I: NADH-Q oxidoreductase; complex II: succinate-Q oxidoreductase; complex III: UQ-cytochrome C oxidoreductase; complex IV: cytochrome C oxidase; and complex V: ATP synthase. It eventually forms ATP through a series of redox processes to provide energy for body tissues. In this assay, related genes on complex I (NDUFA4), complex IV (COX5B, COX6C, COX7C, COX7A2), and complex V (ATP5PO, ATP5F1E, ATP5MC1) were significantly up-regulated in the HS group in both omics. Pigs with abundant energy in their bodies may have a more robust physique, which can be advantageous for securing a strong position in the group competition. These results indicate that the impact of the SGEs on the RFI in the digestive system occurs primarily through substances and energy metabolic pathways.

Furthermore, some reported genes related to social behavior influence the SGEs on the RFI, although the underlying mechanism remains unclear. Apolipoprotein A1 (*APOA1*), the most abundant component of the APOA family and the primary apolipoprotein in lipoprotein, was previously reported to be significantly up-regulated in children and adolescents from high social status and wealthy families, suggesting a possible link between apolipoprotein expression and social ranking [55]. In line with this, we observed that *APOA1*, along with *APOC3* and *APOA4*, was significantly down-regulated in individuals with low SGEs. Fatty acid-binding proteins (*FABPs*) regulate fatty acid absorption and intracellular transport. Studies reveal that there is a close relationship between the FABP family and animal behavior. For instance, *FABP3* knockout mice exhibit reduced social memory and novelty-seeking behaviors, while *FABP7* knockout mice exhibit hyperactivity and anxiety-related phenotypes [56]. This experiment found that both *FABP1* and *FABP2* were significantly up-regulated in the ileum of high-SGE pigs. These findings may point to a potential role for FABPs in behavior regulation, though their functional significance in pigs remains to be explored.

The core genes found in the liver are associated with cholesterol biosynthesis, while those in the ileum are related to cholesterol metabolism. Cholesterol is essential for neuronal development and brain function [57]. Low total cholesterol is widely recognized to be connected with negative psychiatric symptoms such as hostility and impulsivity in severe mental disorders [58], suicide [59], and depression [60]. Disturbed cholesterol homeostasis impairs neuronal survival and susceptibility to excitotoxicity [61]. Cholesterol-rich cells are more resistant to oxidative stress and beta-amyloid toxicity [62]. Therefore, the differences in cholesterol metabolism and maintenance of cholesterol homeostasis could represent one of several possible pathways through which the SGEs may influence the RFI.

## 5. Conclusions

The results from this study indicated that pigs with differing SGE values exhibit distinct feeding patterns. Transcriptomic and proteomic analyses of the liver and ileum identified pathways associated with low- and high-RFI-SGE pigs. Integrated analysis identified potential key genes enriched in mitochondrial processes and oxidative phosphorylation in the liver and in fat digestion and absorption and cholesterol metabolism in the ileum. These findings provide valuable insights into the biological pathways associated with the SGEs and RFI, offering a foundation for future research aimed at improving feed efficiency in the swine industry. Further studies with larger sample size are needed to elucidate the mechanisms underlying this association.

## Figures and Tables

**Table 1:** Variance components from a social genetic model of the RFI.

$\sigma_{A_{d}}^{2}$	$\sigma_{A_{s}}^{2}$	$\sigma_{A_{ds}}$	$r_{A_{ds}}$	$\sigma_{TBV}^{2}$	$\sigma_{l}^{2}$	$\sigma_{g}^{2}$	$\sigma_{e}^{2}$
2594.808 ± 86.02	1007.688 ± 59.78	206.75063 ± 17.32	0.128 ± 0.11	105,451.9714	322.848 ± 19.34	40,354.019 ± 260.16	23,234.581 ± 249.36

$\sigma_{A_{d}}^{2}$, $\sigma_{A_{s}}^{2}$ and $\sigma_{A_{ds}}$ are the additive genetic variance and covariance between the direct effects and social effects. $r_{A_{ds}}$ represents the genetic correlation between the direct and social genetic effects. $\sigma_{TBV}^{2}$ represents the total genetic variance ($\sigma_{TBV}^{2}=\sigma_{A_{d}}^{2}+2\left(n-1\right)\sigma_{A_{ds}}+{\left(n-1\right)}^{2}\sigma_{A_{s}}^{2}$). $\sigma_{l}^{2}$, $\sigma_{g}^{2}$, and $\sigma_{e}^{2}$ represent the random litter, group (pen), and residual variances, respectively. *n* = 10.9 mean pen size, RFI: g/unit.

**Table 2:** Growth performance parameters and feeding behavior of pigs with different SGEs.

Parameter	HS (*n* = 4)	LS (*n* = 4)	*p*-Value
RFI (g)	−248.74 ± 8.70	335.57 ± 4.88	0.001
FCR	1.91 ± 0.11	2.54 ± 0.07	0.001
SGE	10.14 ± 18.85 ^a^	−15.8 ± 18.85	
DGE	−98.92 ± 99.84 ^b^	161.82 ± 99.84	
ADG (g)	971.47 ± 98.31	1000.95 ± 31.53	0.001
ADFI (g)	1856.47 ± 186.73	2541.41 ± 31.29	0.001
AFI (g)	328.36 ± 29.60	296.42 ± 16.86	0.032
NVD	5.66 ± 0.26	8.62 ± 0.40	0.001
TPD (min)	65.83 ± 2.82	50.51 ± 4.22	0.022
DS	76.20 ± 4.66	29.60 ± 5.82	0.001

Results are presented as the mean ± SD, ^a^ and ^b^ denote the HPD95% interval of an SGE and a DGE. RFI: residual feed intake. FCR: feed conversion ratio. SGE: social genetic effect (estimated breeding value). DGE: direct genetic effect (estimated breeding value). ADG: average daily gain. ADFI: average daily feed intake. AFI: average feed intake per visit. NVD: the number of visits to the feeder per day. TPD: total time spent at feeder per day. DS: percentage of displacement success. The *p*-value was determined using Student’s *t*-test.

**Table 3:** Gene information of the top 10 up-regulated and down-regulated genes in the liver in the HS group.

Gene Name	log_2_FC	*p*-Value	FDR	In the HS Group
*TCN1*	5.48	3.14 × 10^−6^	3.31 × 10^−4^	Up
*HBM*	3.77	4.57 × 10^−4^	1.20 × 10^−2^	Up
*HBB*	3.31	2.23 × 10^−4^	7.29 × 10^−3^	Up
*C2H11orf86*	2.73	2.84 × 10^−7^	5.26 × 10^−5^	Up
*LOC100737768*	2.62	9.25 × 10^−4^	1.98 × 10^−2^	Up
*ARF4*	2.54	7.27 × 10^−14^	3.59 × 10^−10^	Up
*PNPLA3*	2.53	3.36 × 10^−5^	1.84 × 10^−3^	Up
*LOC100517779*	2.41	1.75 × 10^−7^	3.45 × 10^−5^	Up
*APOA4*	2.41	4.93 × 10^−7^	8.21 × 10^−5^	Up
*SPATA22*	2.39	1.78 × 10^−7^	3.47 × 10^−5^	Up
*LOC102164346*	−2.43	4.47 × 10^−5^	2.25 × 10^−3^	Down
*CYP1A1*	−2.44	1.99 × 10^−5^	1.22 × 10^−3^	Down
*COLCA1*	−2.46	2.95 × 10^−4^	8.86 × 10^−3^	Down
*LOC110259967*	−2.47	5.12 × 10^−5^	2.47 × 10^−3^	Down
*GALP*	−2.69	8.77 × 10^−4^	1.90 × 10^−2^	Down
*LOC100154757*	−3.01	1.77 × 10^−4^	6.13 × 10^−3^	Down
*KCNH7*	−3.14	1.14 × 10^−4^	4.49 × 10^−3^	Down
*LOC110261964*	−3.24	5.52 × 10^−4^	1.37 × 10^−2^	Down
*ASIC1*	−3.35	1.74 × 10^−4^	6.08 × 10^−3^	Down
*CA3*	−3.88	6.27 × 10^−5^	2.87 × 10^−3^	Down

**Table 4:** Gene information of the top 10 up-regulated and down-regulated genes in the ileum in the HS group.

Gene Name	log_2_FC	*p*-Value	FDR	In the HS Group
*RTL4*	6.09	5.23 × 10^−7^	5.95 × 10^−4^	Up
*ADTRP*	5.43	3.75 × 10^−9^	1.85 × 10^−5^	Up
*SDSL*	5.36	5.81 × 10^−10^	8.59 × 10^−6^	Up
*NTS*	5.29	1.66 × 10^−8^	6.12 × 10^−5^	Up
*KCTD8*	5.24	6.22 × 10^−6^	2.56 × 10^−3^	Up
*FEV*	5.11	6.16 × 10^−4^	2.91 × 10^−2^	Up
*AQP7*	5.04	5.14 × 10^−6^	2.29 × 10^−3^	Up
*MRO*	5.03	7.80 × 10^−4^	3.29 × 10^−2^	Up
*FGFBP1*	5.01	1.20 × 10^−3^	4.20 × 10^−2^	Up
*CXH4orf3*	4.88	2.13 × 10^−9^	1.58 × 10^−5^	Up
*C13H3orf62*	−3.82	2.13 × 10^−4^	1.64 × 10^−2^	Down
*IFNA1*	−3.90	1.8 × 10^−3^	4.94 × 10^−2^	Down
*LOC102165987*	−4.08	7.27 × 10^−4^	3.21 × 10^−2^	Down
*FAT2*	−4.17	1.14 × 10^−3^	4.10 ×10^−2^	Down
*AIRE*	−4.24	1.15 × 10^−3^	4.48 × 10^−2^	Down
*CDH8*	−4.38	1.02 × 10^−3^	3.84 × 10^−2^	Down
*PTPRQ*	−4.82	1.54 × 10^−6^	1.14 × 10^−3^	Down
*ITGB1BP2*	−4.93	1.07 × 10^−3^	3.96 × 10^−2^	Down
*LOC100624648*	−5.89	9.34 × 10^−8^	1.59 × 10^−4^	Down
*GRM8*	−6.10	2.66 × 10^−5^	5.54 × 10^−3^	Down

**Table 5:** DEGs and DEPs with the same expression trend in the HS group.

Name	mRNA-log_2_FC	mRNA-*p*-Value	Pro-log_2_FC	Pro-*p*-Value	Tissue Type
*APOA1*	3.188	0.014	0.278	0.048	ileum
*APOA4*	3.829	0.018	0.397	0.002	ileum
*APOC3*	4.853	0.055	0.337	0.001	ileum
*ASS1*	4.382	0.000	0.473	0.001	ileum
*CDHR2*	2.976	0.001	0.323	0.013	ileum
*DAO*	4.201	0.005	0.367	0.001	ileum
*FABP1*	3.445	0.004	0.598	0.036	ileum
*FABP2*	4.188	0.000	0.839	0.004	ileum
*GSTA1*	5.027	0.016	0.396	0.000	ileum
*LOC100512780*	3.109	0.008	0.363	0.038	ileum
*LOC100738425*	2.239	0.013	0.284	0.010	ileum
*LOC100739663*	4.240	0.024	0.321	0.026	ileum
*LOC106509660*	3.134	0.049	0.295	0.010	ileum
*OAT*	2.964	0.057	0.898	0.049	ileum
*RBP2*	4.029	0.041	0.513	0.002	ileum
*REEP6*	3.219	0.005	0.266	0.000	ileum
*SDSL*	5.510	0.024	0.531	0.000	ileum
*SLC5A1*	3.208	0.001	0.410	0.017	ileum
*STARD4*	2.373	0.014	0.287	0.019	ileum
*ABHD5*	1.133	0.002	0.798	0.000	liver
*ARF4*	2.604	0.000	0.498	0.011	liver
*ARL1*	1.267	0.000	0.734	0.023	liver
*ATOX1*	1.024	0.006	0.266	0.000	liver
*ATP5F1E*	1.147	0.005	0.352	0.000	liver
*ATP5MC1*	2.048	0.000	0.768	0.002	liver
*ATP5PO*	1.138	0.000	0.523	0.000	liver
*CFL1*	1.232	0.000	1.024	0.000	liver
*COX5B*	1.414	0.000	0.867	0.023	liver
*COX6C*	1.456	0.000	0.420	0.002	liver
*COX7A2*	1.632	0.000	0.413	0.023	liver
*COX7C*	1.245	0.000	0.419	0.001	liver
*CYCS*	1.577	0.000	0.304	0.023	liver
*FKBP1A*	1.146	0.000	0.641	0.001	liver
*H2AFZ*	1.127	0.000	0.337	0.048	liver
*HBB*	3.335	0.010	0.284	0.000	liver
*HMOX1*	1.150	0.001	1.082	0.000	liver
*LDHB*	1.354	0.003	0.358	0.001	liver
*MIF*	1.353	0.000	0.489	0.001	liver
*NDUFA4*	1.353	0.000	0.423	0.000	liver
*NNMT*	1.423	0.005	0.332	0.016	liver
*NQO1*	1.279	0.030	0.277	0.005	liver
*PGK1*	1.324	0.000	0.389	0.010	liver
*PSMB6*	1.314	0.000	0.283	0.001	liver
*RTCB*	1.345	0.000	0.343	0.000	liver
*S100A11*	1.063	0.013	1.012	0.000	liver
*SLIRP*	1.333	0.047	0.420	0.007	liver
*TPI1*	1.044	0.000	0.657	0.000	liver
*UBE2D3*	1.857	0.012	0.460	0.002	liver

## Data Availability

All data generated or analyzed during this study are included in this published article and its Supplementary Information Files. The raw transcriptome and proteome data reported in this paper have been uploaded to the NCBI (PRJNA1032745) and iProX database (IPX0007506000), respectively.

## Supplementary Materials

The following supporting information can be downloaded at https://www.mdpi.com/article/10.3390/ani15091345/s1, Supplementary File S1. Animal information and basic data of the HS and LS groups; Supplementary File S2. Transcriptome sequencing data summary and validation; Supplementary File S3. All the differential expression of the mRNA and protein in the ileum and liver; Supplementary File S4. Enrichment analysis of genes and proteins; Supplementary File S5. Cluster dendrogram of modules and module gene counts.
