# Supplementary material for: Transcriptomic and Proteomic Analyses of the Liver and Ileum Identify Key Genes and Pathways Associated with Low and High Groups of Social Genetic Effect of Residual Feed Intake
Source: Animals (Basel). 2025 May 7;15(9):1345. doi: 10.3390/ani15091345 (PMC12070873; doi:10.3390/ani15091345)
Supplement: Supplementary file 1 [file animals-15-01345-s001.zip › Supplementary File S2. Transcriptome sequencing data summary and validation.pdf]

Supplementary Information

Transcriptome sequencing data summary

The transcriptome sequencing data underwent preprocessing steps, including raw data filtering, false discovery rate correction, and GC content distribution examination, resulting in obtaining clean reads for downstream analysis. The data summary is presented in Table S1. The quality of the clean reads meets the requirements for subsequent bioinformatics analysis, with Q20 > 95%, Q30 > 90%, and GC percentage ranging from 40% to 50%.

Table S1 Summary of sample sequencing data quality

Sample	Raw reads (M)	Clean reads (M)	Clean reads ratio	Q20	Q30	GC	Tissue type
LRS1	119.94	117.63	1.93%	96.37	92.12	45.84	Liver
LRS2	119.94	117.91	1.69%	95.75	91.06	44.73	Liver
LRS3	117.00	114.61	2.04%	96.09	91.62	44.46	Liver
LRS4	110.20	107.88	2.11%	96.70	92.72	42.90	Liver
HRS1	119.94	117.29	2.21%	96.37	91.98	45.34	Liver
HRS2	119.94	117.75	1.83%	96.66	92.69	42.02	Liver
HRS3	119.94	115.96	3.32%	96.98	93.21	44.36	Liver
HRS4	119.94	117.87	1.73%	96.81	92.72	46.47	Liver
LRS1	117.44	116.03	1.20%	97.25	93.31	46.32	Ileum
LRS2	108.95	107.30	1.51%	96.10	91.33	44.01	Ileum
LRS3	114.94	113.68	1.10%	96.18	91.43	44.25	Ileum
LRS4	112.06	110.35	1.53%	96.04	91.10	47.07	Ileum
HRS1	119.94	118.04	1.58%	96.18	91.19	47.60	Ileum
HRS2	119.94	118.37	1.31%	96.81	92.19	42.02	Ileum
HRS3	117.44	116.08	1.16%	96.78	92.50	46.51	Ileum
HRS4	112.44	111.27	1.04%	96.17	91.47	47.08	Ileum

Validation of RT-qPCR

To validate the reliability of the RNA-seq results, we randomly selected six genes from the commonly enriched differentially expressed genes (Table S2). These genes were *ABCD3* (ATP Binding Cassette Subfamily D Member 3), *APOA4* (Apolipoprotein A4), *PI16* (Peptidase Inhibitor 16), *RTL4* (Retrotransposon Gag Like 4), *CPXM2* (Carboxypeptidase X, M14 Family Member 2), and *LPIN1* (Lipin 1). Real-time fluorescent quantitative PCR (RT-qPCR) experiments were performed on these genes. The results showed a good agreement between the RT-qPCR and RNA-seq results, indicating the reliability and good reproducibility of the RNA-seq results (Figure 1).

Table S2 Primer sequences and PCR conditions used for RT-qPCR

Name	Primer type	Sequence (5'-3')
GAPDH	upstream	F:5'-CTTCACACGTGCTGATGGAG-3'
	downstream	R:5'-GGAACATGGGTGAGACCTGT-3'
ABCD3	upstream	F:5'-TTCTGTGTCTGCTGCTCACC-3'
	downstream	R:5'-TGGAATTCTGGACCCACTTC-3'
APOA4	upstream	F:5'-TTCTGTGTCTGCTGCTCACC-3'
	downstream	R:5'-TGGAATTCTGGACCCACTTC-3'
PI16	upstream	F:5'-CTGTGAGGCTGCAGTTCTG-3'
	downstream	R:5'-CCTTCTGCACCCACTTTTCC-3'
RTL4	upstream	F:5'-CTGCCTGCTCTCCTTTTCAG-3'
	downstream	R:5'-GGCAGCTGCAGTATCACAAA-3'
CPXM2	upstream	F:5'-ATGAGCCTGCCTGTCAAGAT-3'
	downstream	R:5'-TCTTCTCCACGACATCCACC-3'
LPIN1	upstream	F:5'-CCAGATGTCCCAGTTCCTGT-3'
	downstream	R:5'-TCATCACCCACACTGTCCTC-3'

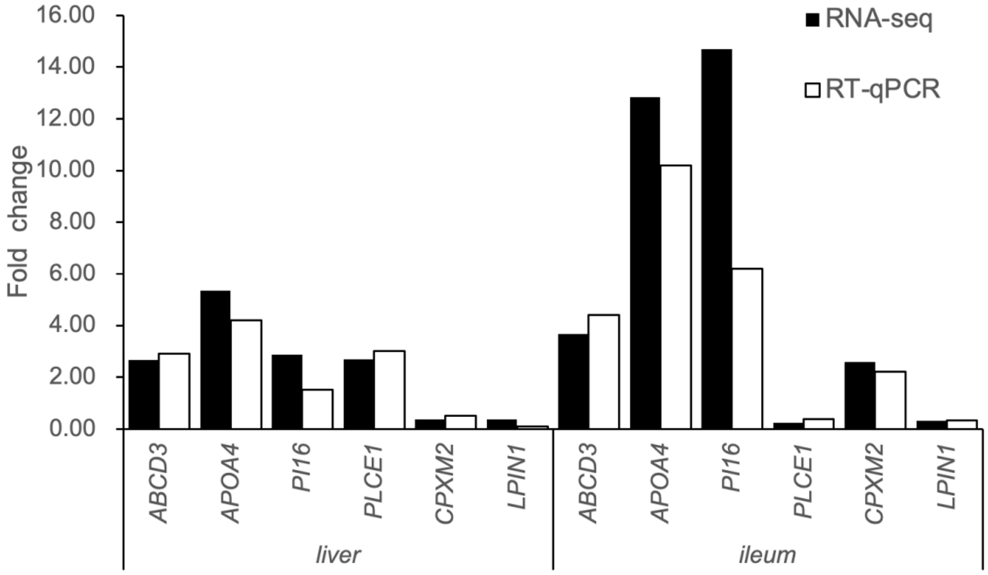

Figure S1 RT-qPCR validation of differentially expressed genes in the liver and ileum tissues
