# Supplementary material for: Transcriptomic and Proteomic Analyses of the Liver and Ileum Identify Key Genes and Pathways Associated with Low and High Groups of Social Genetic Effect of Residual Feed Intake
Source: Animals (Basel). 2025 May 7;15(9):1345. doi: 10.3390/ani15091345 (PMC12070873; doi:10.3390/ani15091345)
Supplement: Supplementary file 1 [file animals-15-01345-s001.zip › Supplementary File S5. Cluster dendrogram of modules and module gene counts.pdf]

Supplementary File S5

Weighted Gene Co-expression Network Analysis

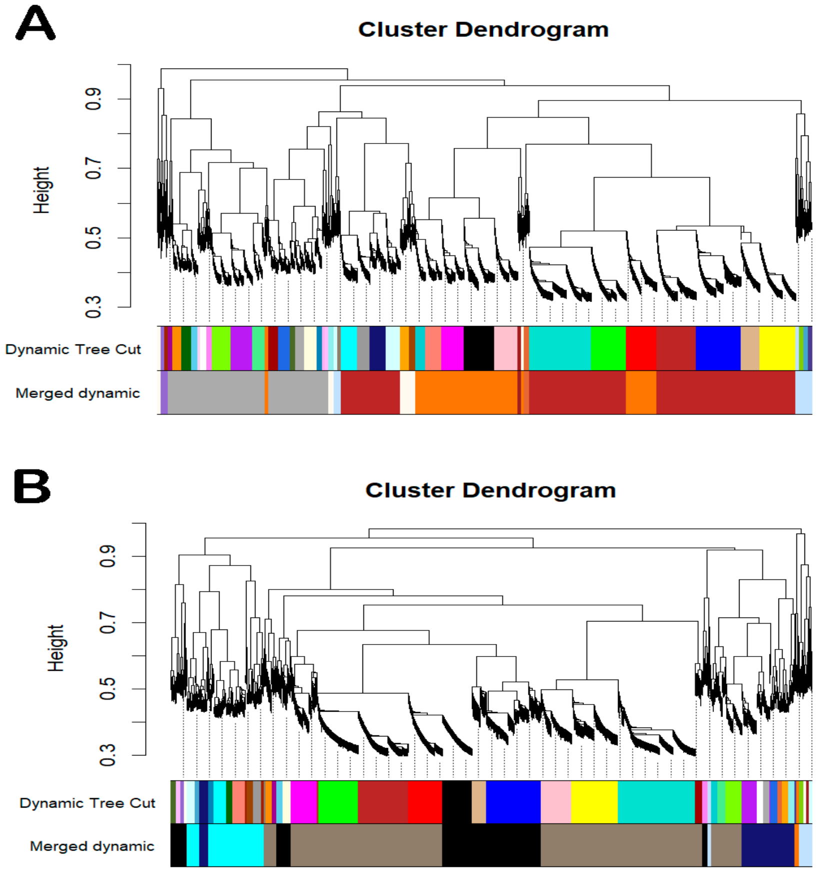

Figure S1. Cluster dendrograms of co-expression modules identified in the liver tissue WGCNA. **(A)** High Social Genetic Effect (HS) group, **(B)** Low Social Genetic Effect (LS) group.

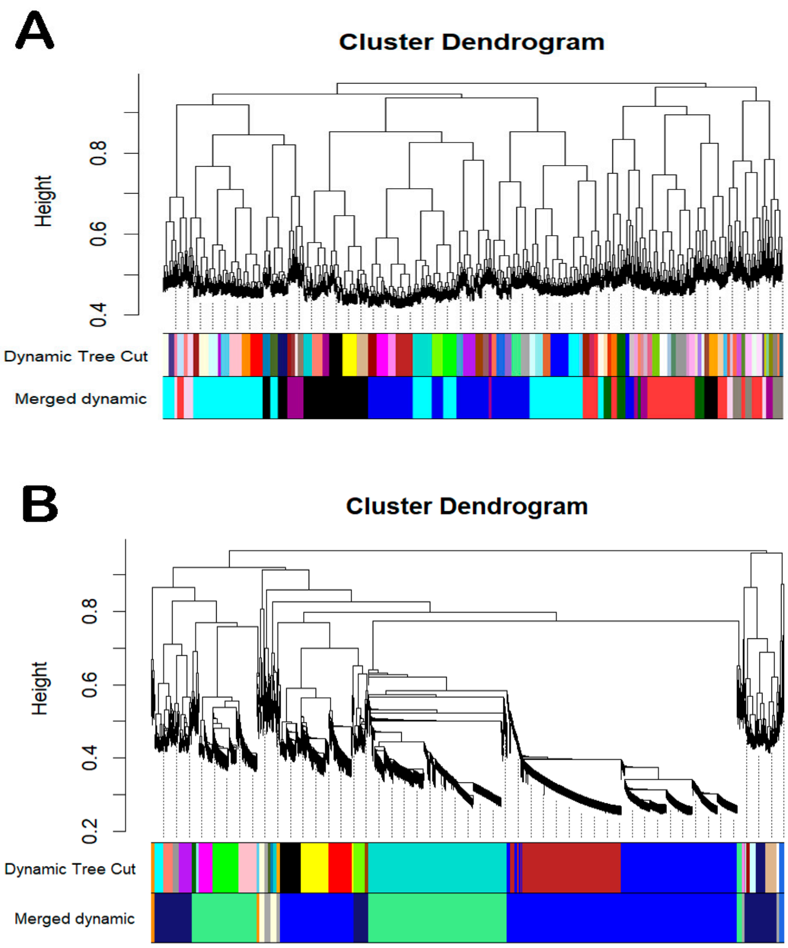

Figure S2. Cluster dendrograms of co-expression modules identified in the ileum tissue WGCNA. **(A)** High Social Genetic Effect (HS) group, **(B)** Low Social Genetic Effect (LS) group.

Table S1. Co-expression modules and gene counts in the liver of the HS group

Module colors	Number
brown	3955
brown4	43
darkgrey	2103
darkorange2	1867
floralwhite	322
lightsteelblue1	300
mediumpurple3	98
sienna3	64

Table S2. Co-expression modules and gene counts in the liver of the LS group

Module colors	Number
bisque4	5406
black	2057
cyan	1035
darkorange2	71
lightsteelblue1	243
midnightblue	939

Table S3. Co-expression modules and gene counts in the ileum of the HS group

Module colors	Number
antiquewhite4	418
black	1640
blue2	2323
brown2	1701
cyan	3114
darkgreen	473
magenta4	617
thistle2	372

Table S4. Co-expression modules and gene counts in the ileum of the LS group

Module colors	Number
blue	4618
darkgrey	228
darkorange	97
lightgreen	3167
lightyellow	174
midnightblue	1281
royalblue	69
